# Alpha-Synuclein Dynamics in Cerebral Ischemia

**DOI:** 10.1080/17590914.2026.2615453

**Published:** 2026-01-29

**Authors:** Sanaz Bordbar, Sina Molavizade, Fateme Dehghani, Samin Davoody, Amir Reza Bahadori, Abbas Tafakhori

**Affiliations:** a School of Medicine, Tehran University of Medical Sciences, Tehran, Iran; b Clinical Research Development Center, Najafabad Branch, Islamic Azad University, Najafabad, Iran; c Student Research Committee, School of Medicine, Shahid Beheshti University of Medical Sciences, Tehran, Iran; d School of Medicine, Shiraz University of Medical Sciences, Shiraz, Iran; e Iranian Center of Neurological Research, Neuroscience Institute, Tehran University of Medical Sciences, Tehran, Iran; f Department of Neurology, School of Medicine, Tehran University of Medical Sciences, Tehran, Iran

**Keywords:** Alpha-synuclein, cerebral ischemia, ischemia, neuroinflammation, regulators, stroke

## Abstract

Cerebral ischemia is defined by insufficient blood supply to the brain and is a leading cause of mortality and neurological disability worldwide. Alpha-synuclein (α-Syn) is a protein associated with several neurodegenerative disorders, including Parkinson’s disease, and has also been linked to the pathophysiology of cerebral ischemia. This narrative review provides a detailed overview of the current understanding of α-Syn in cerebral ischemia. We examine its impact on neuroinflammation, synaptic dysfunction, oxidative stress, and neuronal cell death, as well as its potential protective roles. Additionally, we explore therapeutic strategies targeting α-Syn, including pharmacological agents, gene knockdown models, and RNA-based therapies. We also discuss α-Syn expression changes in animal and human studies and its potential as a diagnostic biomarker. By clarifying the complex interplay between α-Syn and cerebral ischemia, this review aims to deepen our understanding of ischemic brain injury mechanisms and support the development of novel treatment approaches.

## GRAPHIC ABSTRACT

Regulation of α-Syn and its effects after cerebral ischemia. Various modulators regulate α-Syn after cerebral ischemia in both protective and detrimental ways. The left (green) panel highlights the protective roles of microRNAs and the Pep-1-SOD1 complex, which, by acting through multiple pathways, produce positive effects on neural cells and promote recovery following cerebral ischemia. The right (pink) panel shows the roles of PTEN-induced putative kinase 1 (PINK1), polo-like kinases (PLKs), and hyperglycemia, which, through specific known and unknown cellular and molecular pathways, exacerbate neuronal injury and delay brain recovery. Together, these modulators impact α-Syn pathology after cerebral ischemia; however, there are additional known and yet-to-be-identified modulators that are not represented in this figure. Created in BioRender. Davoody, S. (2025) https://BioRender.com/wyem7eq.

## Introduction

Stroke represents a critical and prevalent neurological disorder that affects over 13 million individuals across the world annually. It ranks as the second leading cause of mortality worldwide, following ischemic heart disease, and often presents with sudden and severe symptoms, depending on the region and extent of the affected brain vessels. Almost 87% of all strokes are ischemic and caused by a disruption in cerebral blood flow due to vascular occlusion. Ischemic stroke emerges due to a disruption in cerebral perfusion, mostly resulting from thrombosis or embolism. Following a brief duration, the disruption of blood flow results in oxygen deprivation in the affected brain regions, causing a rapid loss of normal cellular function and usually resulting in irreversible damage in minutes (Orellana-Urzúa et al., 2020). Brain tissue is susceptible to oxidative stress, and even short episodes of reduced blood flow and ischemia can cause cellular damage. This can provoke excitotoxic mechanisms and stimulate the generation of reactive oxygen species (ROS) and inflammation. Ultimately, these processes lead to the death of neuronal cells through both apoptotic and necrotic pathways (Mao et al., 2022; Radak et al., 2014). The reason for this vulnerability is the high utilization of oxygen. Besides, the abundance of polyunsaturated fatty acid chain lipids in brain tissue and the low level of antioxidant enzymes reach the peak of this vulnerability (Friedman, 2010).

Regulation of many proteins is involved in cerebral ischemia, especially through brain cell death (Thiebaut et al., 2019). Synucleins are expressed in neuronal tissue and play a significant role in cerebral ischemia and other neurodegenerative diseases. They are classified into three subtypes: α, β, and γ-synuclein. α-Synuclein (α-Syn) is an ubiquitin protein that is predominantly found in brain tissue, especially at presynaptic terminals, and is a key protein in neurodegenerative diseases. It has antioxidant and neuroprotective action through lipid membrane binding and also plays a role in the modulation of synaptic vesicles (Surguchov, 2015). α-Syn regulates several steps in the synaptic vesicle cycle, such as vesicle clustering, docking, and pool homeostasis (Sharma & Burré, 2023).

Oligomeric α-Syn has a substantial impact on neuronal cell death (Cremades et al., 2012; Winner et al., 2011), and increased levels of α-Syn monomer concentrations can be neurotoxic (Winner et al., 2011). α-Syn also causes pathological changes, for instance, neuronal dystrophy and axonal swelling via the production of Lewy bodies and Lewy neurites in neurons, which end up causing cell death of the neurons (Sung et al., 2001; Volpicelli-Daley et al., 2011). α-Syn is known to be a key protein involved in the pathogenesis of neurodegenerative disorders, particularly Parkinson’s disease (PD) and Alzheimer’s disease (AD)(Bourdenx et al., 2017). Posttranslational modifications (PTMs) of α-Syn have the potential to change its normal function and cause dysfunction in PD and maybe in Lewy body disorders (LBDs) (He et al., 2021; Manzanza et al., 2021). The discovery of the role of α-Syn in multiple neurodegenerative disorders has led to the establishment of α-synucleinopathies as a noteworthy class of neurodegenerative diseases (Goedert, 2001). In focal cerebral ischemia, α-Syn expression and its movement to the nucleus are regulated in rodent brain neurons (Kim et al., 2016). In addition, notable reductions in infarction, oxidative stress, and apoptosis, and improved neurological recovery have been observed with the knockdown or knockout of α-Syn in rodents (Chelluboina et al., 2020). In contrast, evidence from some studies reveals that α-Syn has neuroprotective effects, protecting against oxidative stress and cellular injury while inhibiting apoptosis and regulating dopamine at synapses (Albani et al., 2004; Sidhu et al., 2004). Moreover, cerebral ischemia has been reported to reduce the expression of α-Syn in brain tissue, which may aggravate brain damage (Kang et al., 2018). We performed a thorough review of the role of α-Syn in ischemic stroke and its associated pathophysiological mechanisms, uncovering its potential mechanisms to adapt to more effective therapeutic interventions.

## Alpha-Synuclein

### Structure

The term α-Syn is derived from its cellular distribution: “syn” from the synapse and “nuclein” from the nucleus. α-Syn is a small cytosolic protein, weighing 19 kDa and composed of 140 amino acids (Burré, 2015; Burré et al., 2018). It is encoded by the SNCA (Synuclein Alpha) gene, located in the long arm of chromosome 4, and it has three distinct areas: a positively charged, N-terminal lipid-binding domain forms an amphipathic helical structure upon binding to detergent micelles or phospholipid vesicles; a central hydrophobic area referred to as the NAC (an acronym resulting from the names of the three genes, NAM (no apical meristem), ATAF1,2 and CUC2 (cup-shaped cotyledon) domain (for the non-Aβ part of senile plaques) that may be pivotal in the oligomerization and aggregation of synucleins, as it has the potential to form cross β-structures.; and a highly acidic C-terminal domain (Fusco et al., 2014).

### Physiology

In adults, α-Syn is primarily found in the central nervous system (CNS), particularly in presynaptic terminals, and constitutes almost 1% of the soluble proteins in the brain (Recchia et al., 2004). In the absence of chemical cross-linkers and oligomer-promoting substances like dimethyl sulfoxide, monomeric α-Syn was identified as the predominant form present in the cytoplasm (Dettmer et al., 2013). α-Syn is expressed in different cellular structures, including synaptic vesicles, inner mitochondrial membrane, mitochondria-associated endoplasmic reticulum membrane, the Golgi apparatus, and endosomes (Bernal-Conde et al., 2019). Although there is limited evidence, α-Syn plays an essential role in the integrity of synaptic vesicles and presynaptic function in primary hippocampal neurons, thereby contributing to their homeostasis and neurotransmitter release (Cheng et al., 2011; Sulzer & Edwards, 2019). Furthermore, α-Syn participates in every aspect of synaptic vesicle cycling, consisting of the regulation of the size of the vesicle pool, mobilization, and endocytosis.

α-Syn acts as an antioxidant by interacting with lipid membranes, where its methionines are oxidized by hydrogen peroxide, reducing lipid oxidation (Zhu et al., 2006). α-Syn repeat sequences are key contributors to the disruption of lipid bilayers. They cause a helical conformation in α-Syn, which prevents its propensity towards β-structures (Zhu et al., 2003). Based on the promoter type, its strength, and the brain region where the protein is expressed, increasing α-Syn levels leads to varying effects and phenotypes (Maskri et al., 2004).

Although debated, α-Syn has been found in the nucleus. The N- and C-termini of a-syn participate in nuclear translocation and can increase its nuclear localization through familial mutations, PTMs, and oxidative stress (Goers et al., 2003). The precise mechanism through which nuclear α-Syn influences transcription is still uncertain; however, it likely involves direct DNA interactions or the modulation of essential transcriptional regulators (Davidi et al., 2020; Surguchov, 2023). α-Syn interacts with histones, where it can regulate their activity in cycles of acetylation and deacetylation, which are likely dependent on α-Syn levels (Paiva et al., 2017; Surguchov, 2023). α-Syn predisposes to various PTMs, which likely affect its physiological or pathological functions (Canever et al., 2023). Phosphorylated α-Syn is highly and selectively present in α-Syn deposits across different synucleinopathies. This adjustment has been reported to influence α-Syn aggregation (Fujiwara et al., 2002; Shan et al., 2021). As well, C-terminal domain interactions with the NAC region of α-Syn also inhibit its aggregation (Herrera et al., 2008).

Following cerebral ischemia, transcriptional and post-translational changes occur in α-Syn that shift it toward pathogenic forms. To illustrate, ischemic stress triggers the expression of SNCA and promotes the movement of α-Syn into the nucleus, where it can interact with chromatin and affect transcriptional activities (Kim et al., 2016). At the same time, the activation of kinases such as Polo-like kinases enhances phosphorylation at serine-129, which is closely associated with protein misfolding and aggregation (Fujiwara et al., 2002). Additional ischemia-associated modifications, including but not limited to nitration and truncation, further reduce α-Syn stability and promote oligomer formation (He et al., 2021). These oligomeric species disrupt mitochondrial respiration, elevate oxidative stress, and impair calcium and autophagic homeostasis, thereby amplifying neuronal vulnerability after ischemia (Angelova & Abramov, 2024).

### Alpha-Synuclein Aggregation in Synucleinopathies and Neurodegenerative Disorders

Protein misfolding and aggregation are hallmark characteristics of neurodegenerative disorders and are characterized by the presence of Lewy bodies and proteinaceous aggregates, which are primarily composed of α-Synuclein (Pietrobono et al., 2017; Soto & Pritzkow, 2018). α-Syn misfolding and aggregation disrupt regular cellular function to result in the development of cytoplasmic inclusion bodies in affected brain regions. This accumulation contributes to the development of bodies within affected brain regions. It contributes to the development of synucleinopathies, including PD, LBD, and multiple system atrophy (Estaun-Panzano et al., 2023). The accumulation of α-Syn in PD brainstem and cortical regions leads to motor dysfunction and cognitive impairment (Kim, 2013). Furthermore, missense mutations and gene duplications in the SNCA gene encode α-Syn and are closely related to autosomal dominant familial PD (Simitsi et al., 2020; Xu et al., 2015). Interestingly, PD patients exhibit elevated α-Syn levels in peripheral blood mononuclear cells compared with healthy individuals. α-Syn accumulates in senile plaques and limbic regions and is responsible for AD pathology. α-Syn has the potential to be implicated in tau and amyloid-beta protein fibrilization and potentially worsening cognitive impairments (Crews et al., 2009; da Costa, 2003; Shan et al., 2021). The role of α-Syn and its aggregation in previous neurodegenerative disorders provides notable insights into the function of α-Syn in cerebral ischemia.

## Potential Pathophysiological Role of α-Syn in Ischemia

Various intra- and extracellular pathophysiological processes involving α-Syn following cerebral ischemia have been described. Additionally, potential roles of α-Syn aggregation, similar to those seen in neurodegenerative diseases, have been investigated to enhance our understanding of α-Syn mechanisms in cerebral ischemia.

### Mitochondrial Impairment

Aggregation and overexpression of α-Syn significantly contribute to mitochondrial injury and oxidative stress in the neurons. It elevates ROS levels, enhances protein tyrosine nitration, and thus impairs cellular respiration (Parihar et al., 2009). The overproduction of ROS and reactive nitrogen species (RNS) oxidizes mitochondrial proteins, lipids, and DNA, which further causes mitochondrial injury and increases cellular damage (Bhat et al., 2015). α-Syn oligomers accumulate in mitochondria and exert their destructive effects on ATP synthase by reducing complex I-dependent respiration (Ludtmann et al., 2018). Additionally, it induces oxidative stress, which opens the mitochondrial permeability transition pore, thereby destroying mitochondrial function and ultimately end up in cell death (Chinta et al., 2010; Tryphena et al., 2023). It must be pointed out that the current complex I-deficient cells would be less sensitive to subsequent mitochondrial injury by α-Syn (Reeve et al., 2015). Furthermore, the N-terminal region of α-Syn possesses a mitochondrial-targeting signal that may enable it to interact with the inner membrane (Devi et al., 2008). These combined observations point to an important role for mitochondria, particularly complex I, in the damage due to α-Syn.

### Inflammation

After ischemia induced by middle cerebral artery occlusion (MCAO), immune activity within neuronal tissue increases markedly. Afterwards, the production of ROS increases and then triggers the elevation of α-Syn in the brain (Musgrove et al., 2019). Also, the extracellular deposition of α-Syn results in neuroinflammation by activating microglia and following dopaminergic neurodegeneration (Zhang et al., 2005). In addition, α-Syn protofibrils stimulate the p38 and ERK1/2 mitogen-activated protein kinases (MAPKs) cascades and the NF-κB signaling pathway in microglia, resulting in neuronal cell loss (Wilms et al., 2009). Hoenen et al. presented evidence that the mutant form of α-Syn, A53T variant, induces more potent microglia activation than wild-type α-syn. This increased activation is through several important signaling pathways, i.e., MAPK, NF-κB, Activator Protein 1 (AP-1), and Nuclear factor erythroid 2-related factor 2 (Nrf2) (Hoenen et al., 2016). According to Lohmann et al., neuroinflammation increased in the early phase of ischemia following MCAO in a familial A53T mutant mouse model that expresses human α-syn, followed by a reduction in the neuroinflammatory response thereafter (Lohmann et al., 2022). The initial elevated immune response likely represents a typical reaction to ischemic events (Pluta et al., 2021). Lohmann et al. later noted another phase of the immune response occurring between 180 and 360 days post-MCAO induction, which they hypothesized was directed at clearing pathological α-Syn deposits and the associated neuronal loss (Lohmann et al., 2022). A previous experiment explored the influence of Toll-like receptor 4 (TLR4) on astroglial and microglial responses to α-Syn accumulation. In wild-type TLR4 cells, there was a marked elevation in the production of proinflammatory cytokines. These cells also showed an improved ability to phagocytose, and they generated higher levels of ROS compared to TLR4-null cells (Fellner et al., 2013). This study highlights the important role that TLR4 plays in how microglia and astroglia respond to α-Syn, as it reveals that TLR4 can be a regulator of both inflammatory and phagocytic activity. This experiment suggests that TLR4 plays a significant role in microglia and astroglia reaction towards α-Syn, especially in the regulation of inflammatory and phagocytic activity. So these findings confirm the hypothesis that α-Syn is involved in neuroinflammation following an ischemic injury. But it is not clear whether the amplified immune reaction is involved in the deposition of aggregated α-Syn or has any effect on its pattern of deposition.

### Lipid Peroxidation

Lipid peroxidation can lead to neural damage (Block et al., 1995; Kuribayashi et al., 1992). Additionally, α-Syn plays a role in lipid peroxidation (Angelova et al., 2015; Bonini & Giasson, 2005; Cremades et al., 2012). Lipid peroxidation leads to disturbances in neuron permeability, metabolism, and ion transport (Nigam & Schewe, 2000). A previous study that examined the role of α-Syn in induced MCAO evidenced that lowering α-Syn levels enhances lipid peroxidation. α-Syn plays an antioxidant role against lipid oxidation in such a way that, at first, it binds to lipid membranes, and then, hydrogen peroxide oxidizes to sulfoxide four of α-Syn methionines (Zhu et al., 2006). On the other hand, it has been shown that ROS leads to lipid peroxidation by binding to membrane phospholipids (Kleikers et al., 2012; Lee et al., 2012). Lipid peroxidation is considered a pathological mechanism of cerebral ischemic injury (Adibhatla & Hatcher, 2006). Angelova et al. demonstrated that the accumulation of α-Syn in a β-sheet acts as a toxic oligomeric form, elevating ROS production and leading to lipid peroxidation (Alessandri et al., 2004; Deas et al., 2016). It is also possible that α-Syn oligomers form pore-like and annular complexes in the lipid membrane, thereby disrupting calcium homeostasis (Danzer et al., 2007; Stefanovic et al., 2014; van Rooijen et al., 2010). In addition, related to mitochondrial disruption, the mechanism of mitochondria involves fusion and fission dynamics. Disturbances in this pathway in neurons lead to neurodegenerative disorders (Chen & Chan, 2009). Lipid peroxidation leads to mitochondrial damage, so ROS production increases, and oxidative stress is induced (Green & Reed, 1998).

### Impaired Synaptic Function

One of the presynaptic proteins is α-Syn, which regulates synaptic vesicle recycling and maintains the balance of the recycling pool. While its exact role is still debated, it is essential for neurotransmitter release and the stability of synaptic vesicle pools (Scott & Roy, 2012; Sharma & Burré, 2023). Excessive α-Syn can initiate a pathological cascade that leads to the depletion of essential presynaptic proteins involved in both exocytosis and endocytosis. Consequently, neurotransmitter release is affected, as well as an enlargement in synaptic vesicle size, leading to synaptic dysfunction (Scott et al., 2010). Importantly, even acute increases in α-Syn levels can heavily disrupt synaptic vesicle recycling back from the plasma membrane, with the N-terminal α-helical domain (Busch et al., 2014). Understanding these molecular physiopathologies helps understand the study and modify the effects of α-Syn aggregation on the dysfunction of synapses after cerebral ischemia.

### Calcium Dysregulation

Prior research indicates that α-Syn aggregation significantly contributes to neuronal calcium dysregulation (Angelova et al., 2016). Both oligomeric and monomeric α-Syn can integrate into cellular membranes and induce calcium signaling within neurons and astrocytes (Angelova et al., 2016). When intracellular calcium levels rise, potentially because of mitochondrial dysfunction or calcium channel imbalances, it can facilitate α-Syn aggregation (Rcom-H’cheo-Gauthier et al., 2016). Furthermore, α-Syn aggregates interface with oxidized lipid membranes, disrupting calcium balance and activating ferroptosis, which is a kind of cell death driven by iron (Angelova et al., 2020). Mutually, elevated calcium levels and oxidative stress promote neuronal aggregation of α-Syn (Rcom-H’cheo-Gauthier et al., 2014). This evidence suggests that calcium dysregulation and α-syn aggregation together initiate a destructive cycle following neural ischemic events. This cycle may progressively exacerbate cellular damage, potentially resulting in neurotoxicity and the onset of neurodegenerative diseases.

### Autophagy Impairment

Autophagy is a conserved cellular degradation pathway that removes damaged organelles, misfolded proteins, and toxic cellular components through lysosomal processing. It plays a crucial role in maintaining cellular homeostasis, especially under conditions of stress or nutrient deprivation. Tanik et al have shown that α-Syn aggregates hinder degradation and can disrupt macroautophagy by delaying the maturation of autophagosomes (Tanik et al., 2013). These aggregates remain present even when soluble α-Syn levels decrease, which shows resistance to removal. Thus, the impairment of cellular protein degradation and overexpression of α-Syn systems accelerate its aggregation (Bellomo et al., 2020). The relationship between α-Syn aggregation and disablement of the autophagic-lysosomal system seems to be bidirectional, with each pathway worsening the other in a vicious cycle (Bellomo et al., 2020). Elucidation of this interaction could offer new therapeutic strategies for the recovery of autophagy, with potential therapeutic implications for α-Syn aggregation following neuronal ischemic events.

### Role of Tau Protein

Tau protein plays an important protective role for microtubules and is essential for the polarity of neurons, vesicular transport, and signal transduction (Rankin et al., 2007). Cerebral ischemia can typically cause the tau protein to become hyperphosphorylated and can lead to cell death in neurons (Chen & Jiang, 2019; Wen et al., 2004). Mehta et al. studied the relationships between Glycogen synthase kinase 3β (GSK-3β), a key driver of tau phosphorylation, α-Syn, and tau phosphorylation following brain ischemia. Firstly, they figured out that brain ischemia in mice increases α-Syn binding and phosphorylated tau levels. They reported that in α-Syn knockout mice, GSK-3β and tau protein remained unchanged after ischemia, which seemed to prevent tau from becoming phosphorylated. Also, Tau protein phosphorylation can be inhibited by administering a GSK-3β inhibitor. Fascinatingly, neuronal ischemia caused notably less neuronal impairment in tau knockout mice (Mehta et al., 2023). These evidences signify the involvement of the tau protein and GSK-3β in the postischemic phase of brain ischemia and their interaction with α-Syn, suggesting them as potential future therapeutic targets after stroke (Figure 2).

## Positive Role of α-Syn in Cerebral Ischemia

The role of α-Syn has not been explored in all studies, as most studies have explored its role in the pathophysiology of many disorders. However, certain studies have simplified its role as a protective agent. Here, we discuss **the** findings of these studies in detail. *Koh* et al. reported that low α-Syn levels can cause pathology, indicating its protective role. The authors measured α-Syn levels via western blotting following cerebral ischemia in a mouse MCAO model. α-Syn reportedly decreases following ischemia. These results indicate that low measures of α-Syn might cause cytotoxicity. Additionally, glutamate treatment reduced the amounts of α-Syn, therefore causing neural death. Therefore, a reduction in α-Syn can cause neural death and brain damage (Koh, 2017). These findings indicate that its role in neural survival is related to the Phosphoinositide 3-kinase/Protein Kinase B (PI3K/Akt) pathway and regulating B-cell lymphoma 2 (Bcl-2) protein (Seo et al., 2002). In addition to its role in the pathologies of disorders, α-Syn has a role in neural maturation. *Taguchi* et al. investigated α-Syn in juxtaglomerular neurons (JGNs), which highly express α-Syn in normal conditions in the olfactory bulb. α-Syn expression responds to ischemia. Sex-determining region Y-box 2 (Sox2), which plays a role in neural immature identity, is highly expressed in most α-Syn-enriched JGNs. In α-Syn homozygous knockout mice, the count of Sox2-positive JGNs was considerably greater than that in heterozygous knockout mice. They induced cerebral ischemia in wild-type mice and homozygous knockout mice. Sox2-positive JGNs and Sox2 in α-Syn-enriched JGNs decreased considerably based on the expression of α-Syn. The expression of neural nuclei (mature neural markers) was increased in αSyn-enriched JGNs. A decrease in the number of Sox2-positive JGNs induced by cerebral ischemia was not detected in Syn homozygous knockout mice. Overall, α-Syn has been proven to play a role in the maturation of JGNs in the olfactory bulb of mice (Taguchi et al., 2020). Overall, we can deduce that α-Syn is not all pathological and can play certain positive roles. It has a dual role in neural pathology and maturation. A decrease in α-Syn can cause cytotoxicity and neural death via the PI3/Akt pathway and Bcl-2 regulation. Moreover, α-Syn influences the maturation of the olfactory bulb’s JGNs, with alterations in Sox2 expression linked to α-Syn levels, suggesting its critical role in neuronal identity and response to ischemic conditions.

## Regulators of α-Syn in the CNS

### Pep-1-SOD1

Protein Transduction Domain 1 (PEP-1) fused with Superoxide Dismutase 1 (SOD1), a fusion protein that combines the cell-penetrating peptide Pep-1 with SOD1, has been shown to possess a protective effect on neuronal cells in cerebral ischemia (Hwang et al., 2005). One study assessed the effect of Pep-1-SOD1 on α-Syn accumulation following ischemia and figured out that gerbils treated with Pep-1-SOD1 exhibited significant reduce of α-Syn levels in the hippocampal CA1 region compared to the control group. In addition, they found that age can influence α-Syn levels, with greater accumulation in aged gerbils compared to adults (Hwang et al., 2005).

### Hyperglycemia

Hyperglycemia is linked to a greater risk of complications and even death in patients experiencing an acute ischemic stroke (Ferrari et al., 2022). Chronic hyperglycemia has been seen to cause α-Syn aggregation, ultimately resulting in degeneration of dopaminergic neurons and increased neuroinflammation, and accelerates the progression of PD (Lv et al., 2021; 2022). It has been concluded that although α-Syn aggregation was more significant in nondiabetic mice, diabetic mice exhibited larger neurological deficits and increased infarct volume (Kang et al., 2018). This effect of diabetes on α-Syn differs from what earlier studies on neurodegenerative diseases have shown and warrants more exploration, particularly after ischemic stroke.

### Polo-like Kinases

In normal physiological circumstances of neurons, α-Syn remains unphosphorylated within the cell (Kawahata et al., 2022). The phosphorylation of α-Syn is mediated primarily by kinases, especially Polo-like kinases (PLKs). One of the important modulators of mitosis and the cell cycle in eukaryotic cells is PLK1, which has been specifically identified as having a high affinity for the S129 site of α-Syn and acts as a key regulator in controlling the extent of α-Syn phosphorylation (Archambault & Glover, 2009; Inglis et al., 2009; Mbefo et al., 2010; Salvi et al., 2012). Kim et al. performed cerebral ischemia in PLK2 knockout mice and observed that the size of infarct volume was significantly decreased, and both functional recovery and neurological restoration were better in these knockout mice compared to the wild-type PLK2 mice. These observations imply that cells lacking PLK2 display enhanced resistance to neuronal damage, and the S129 phosphorylation of α-Syn is the pathogenic aftermath of neuronal ischemia (Kim et al., 2016). These findings suggest that targeting PLK2 may help prevent its ability to upregulate α-syn.

### MicroRNAs

Previous studies have shown that miRNA-7 and miRNA-153 bind specifically to the 3’ untranslated region of the α-Syn mRNA, which contributes to reducing protein levels through post-transcriptional regulation (Doxakis, 2010; Junn et al., 2009). Additionally, additionally facilitates autophagy-mediated degradation of α-Syn aggregates (Choi et al., 2018). Furthermore, it has been observed that miRNA-7 plays a neuroprotective function against oxidative stress by downregulating α-Syn expression (Junn et al., 2009).

### PTEN-Induced Putative Kinase 1

**Figure 1.**
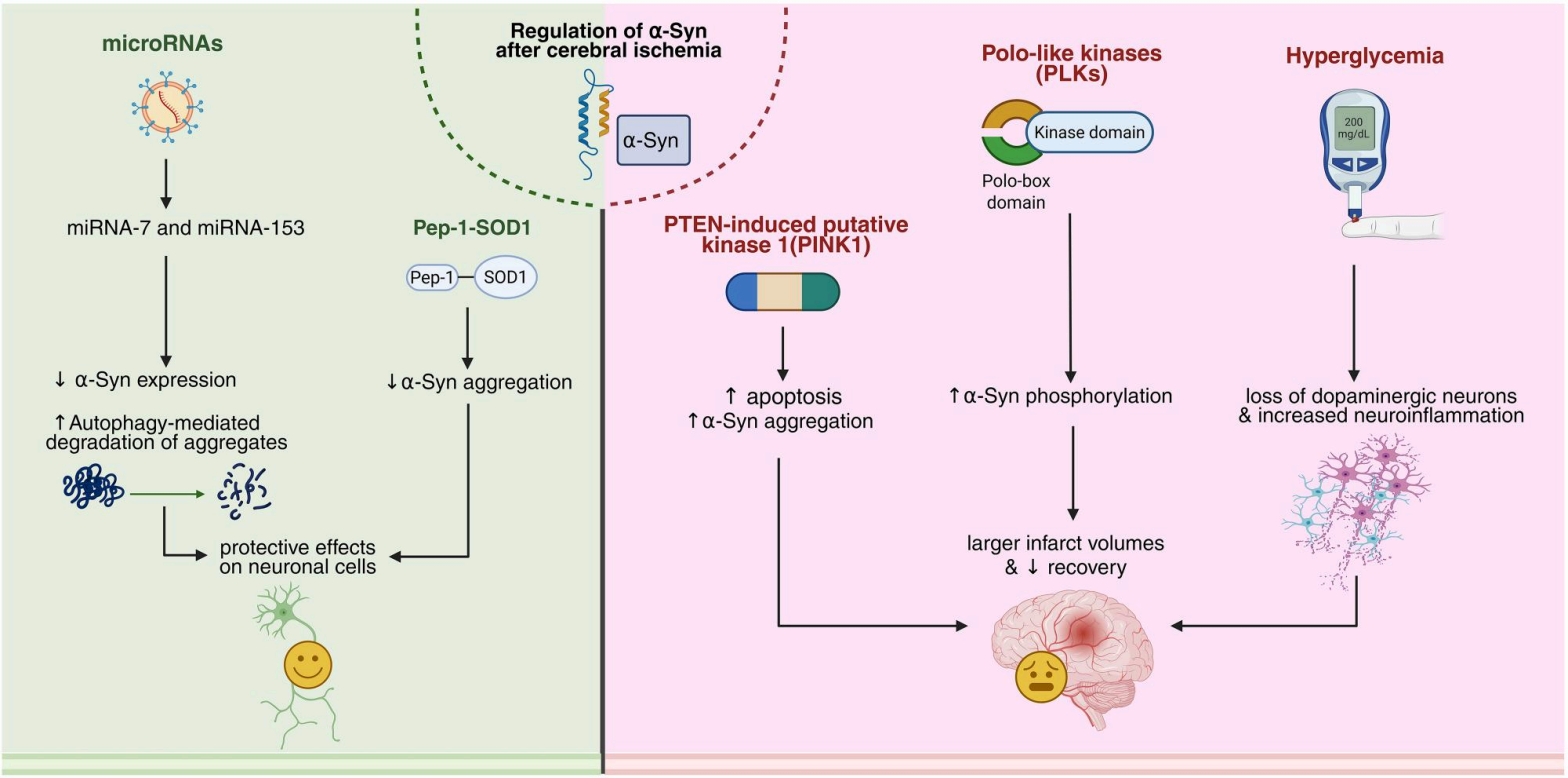
Regulation of α-Syn and its effects after cerebral ischemia. Various modulators regulate α-Syn after cerebral ischemia in both protective and detrimental ways. The left (green) panel highlights the protective roles of microRNAs and the Pep-1-SOD1 complex. MicroRNA-7 and microRNA-153 reduce α-Syn levels by suppressing its mRNA translation and promoting autophagy-mediated degradation, thereby indirectly decreasing α-Syn aggregation and supporting neuronal recovery following cerebral ischemia. The right (pink) panel shows the roles of PTEN-induced putative kinase 1 (PINK1), polo-like kinases (PLKs), and hyperglycemia, which, through specific known and unknown cellular and molecular pathways, exacerbate neuronal injury and delay brain recovery. Together, these modulators impact α-Syn pathology after cerebral ischemia; however, there are additional known and yet-to-be-identified modulators that are not represented in this figure. Created in BioRender. Davoody, S. (2025) https://BioRender.com/wyem7eq.

PTEN-induced kinase 1 (PINK1) is a mitochondrial serine/threonine-protein kinase that is deduced to guard cells against stress from mitochondrial dysfunction. A study aimed to investigate PINK1’s negative effect on the brains of PD-10 mice subjected to hypoxia and ischemia. They first induced ischemia in PINK1-knockout mice. These authors reported that, compared with wild-type mice, knockout mice presented decreased apoptosis and, as a result, less brain infarction. Additionally, the amount of α-Syn was measured via western blotting, and α-Syn amount was significantly increased in the knock-out group than that in the control. The levels of α-Syn were decreased by small interfering RNA (siRNA); the results were completely reversed, and the degree of brain damage increased. This study revealed the neuroprotective effects of α-Syn and its opposite relationship with the expression of PINK1 (Zhu et al., 2016) (Figure 1).

**Figure 2.**
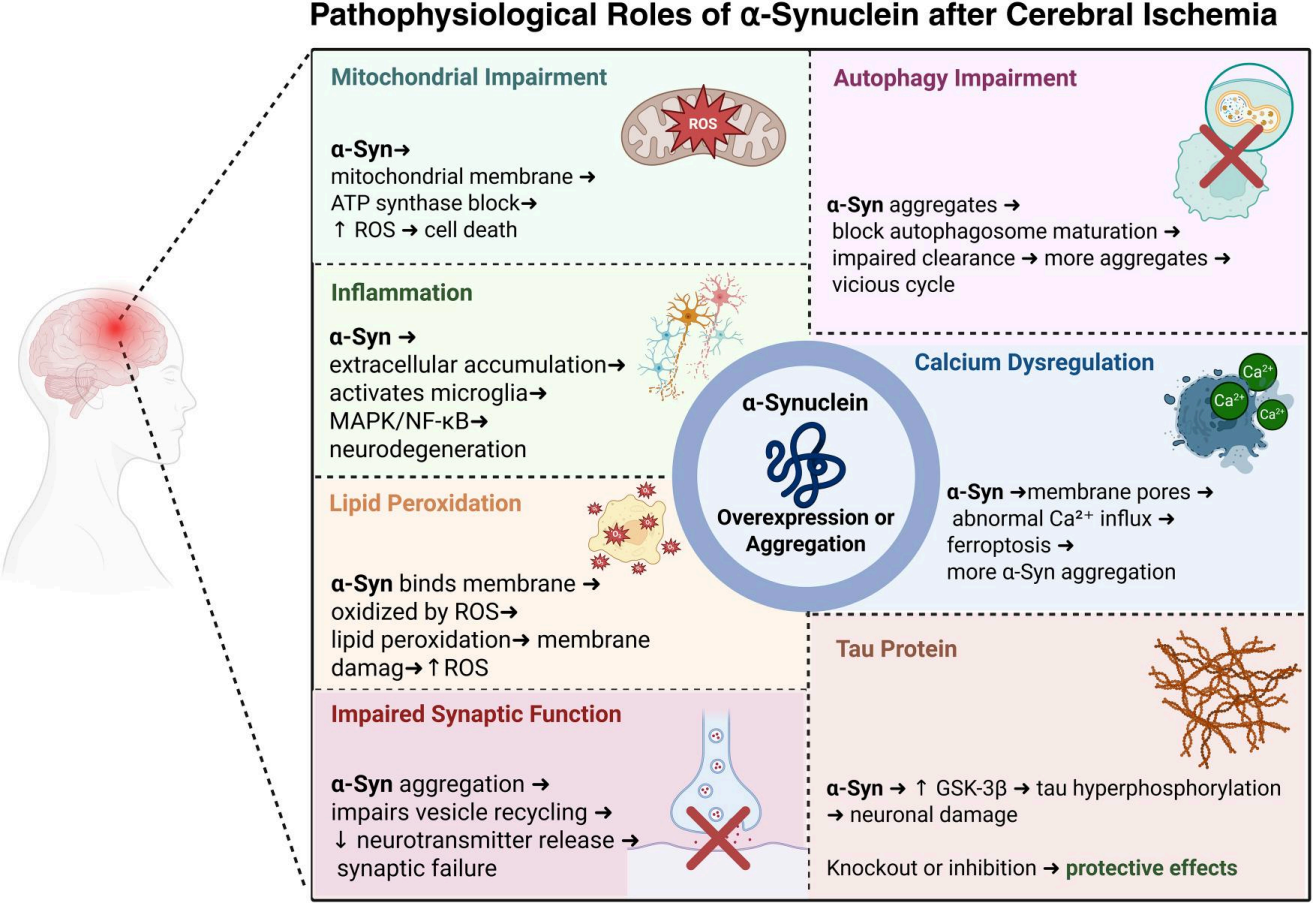
Pathophysiological roles of α-Synuclein after cerebral ischemia. Alpha-synuclein (α-Syn) following cerebral ischemia is strongly linked to various cellular/molecular mechanisms. In brief, these include but are not limited to: mitochondrial impairment, neuroinflammation, lipid peroxidation, impaired synaptic function, calcium dysregulation, autophagy impairment, and tau hyperphosphorylation. Created in BioRender. Davoody, S. (2025) https://BioRender.com/5j2bcmr.

## Changes in Alpha-Synuclein Expression Levels following Cerebral Ischemia

In this section, we summarize research on α-Syn expression following cerebral ischemia, with studies focusing primarily on protein expression, post-translational modifications (such as phosphorylation and aggregation), and cellular localization, rather than gene transcriptional regulation. These investigations highlight how ischemic stress alters α-Syn dynamics in both animal models and human patients, providing insight into its role in neurodegeneration.

### Animal Models

Multiple studies have researched the expression level of α-Syn after cerebral ischemia through different means. Here, we discuss **the** findings of these studies in detail.

*Ischimaru* et al. first measured changes in precursors of the non-beta compartment of amyloid (NCAP), which is α-Syn, following ischemia in bilateral common carotid artery occlusion (BCAO). NCAP immunoreactivity was observed in the hippocampus after ischemia in the Cornu Ammonis 1 (CA1)region. NACP-positive structures were present following ischemia. They reported that NCAP has a role in ischemia (Ishimaru et al., 1998).

*Yu* et al. investigated the effect of a monoclonal antibody produced for the detection of α-Syn. They measured **the** effects of acute hypoxia on α-Syn expression in the cerebral cortex. They reported that α-Syn increased after the first hypoxia but then decreased to basal levels after the fourth hypoxia. They reported that α-Syn-immunopositive substances were based in the same neurons’ nuclei in addition to the nerve endings. The density of neurons with α-Syn-immunopositive nuclei increased **and** then decreased to normal levels over time. They reported that neurons with α-Syn-positive nuclei are affected by hypoxia. These authors reported that there is a need for more research on the mechanisms involved (Yu et al., 2004).

*Yoon* et al. investigated the immunoreactivity and α-Syn in the hippocampus of gerbils in a model of transient BCAO. They reported that immunoreactivity increased following ischemia. They also reported that the levels increased in the CA1 region of the hippocampus. α-Syn was more prevalent in the aged group than in the adult group. However, with time, it starts to decrease (Yoon et al., 2006). Like *Yoon* et al., *Hu* et al. measured α-Syn levels following ischemia via common carotid occlusion. They reported that its level increases following ischemia and is related to dopamine metabolism (Hu et al., 2006).

*Similar to previous studies, Unal-Cevik* et al. reported **an** increased amount of measured α-Syn following ischemia. They measured α-Syn levels in transgenic mice with MCAO. They reported that ischemia increases the amount, oligomerization, and then aggregation of wild-type α-Syn. In alphaSYN transgenic mice, the infarct area was larger, with more α-Syn fibrils. They reported that the aggregation is associated with oxidative stress. Its aggregation occurs during ischemia, also decreases neural survival, and its misfolding is neurotoxic. Transgenic mice are relatively sensitive to ischemia and neural necrosis (Unal-Cevik et al., 2011). Similar to these studies*, Kim* et al. reported the same finding regarding α-Syn in a model of transient focal ischemia. Ischemia plays a role in the nuclear translocation of α-Syn. They reported that serine-129 (S129) phosphorylation of α-Syn (pα-Syn), in addition to oligomerization of α-Syn, was increased following ischemia. Oligomerization can be considered a hallmark of synucleinopathies and causes aggregation. Additionally, pα-Syn nuclear translocation was increased. Additionally, α-Syn monomers cause aggregation following ischemia (Kim et al., 2016).

*Finally, Lohmann* et al. investigated the changes in a certain mutated α-Syn. They investigated the effects of cerebral ischemia in TgM83 mice, which express α-Syn having the A53T mutation. The mice did not show any neuropathology or neurologic signs. Motor deficits were observed six months postischemia and worsened until 12 months. Neural loss was observed in the infarct area via immunohistochemical analysis. Inflammatory signs, including microgliosis and astrogliosis, were observed. Inflammation and the infarct area decreased gradually 180 days post-surgery, but inflammation and neural loss increased after 360 days. Sham-treated mice presented no signs of neuropathology. Sham mice did show a noteworthy change in the level of α-Syn. The amount of α-Syn after surgery decreased, but after 180 days, it started to increase and remained below the normal level. These authors stated that increased oligomerization and aggregation might be the reason for this difference. An increase in α-Syn, aggregation, and intracellular deposits was observed. α-Syn reportedly colonizes microglia, neurons, and oligodendrocytes. Subsequently, a loss of dopaminergic neurons in the substantia nigra was observed. They revealed a link between cerebral ischemia and PDs (Kim et al., 2016).

Overall, we can deduce that α-Syn levels tend to increase following ischemia, especially in response to hypoxia, before decreasing over time. However, only **a** few studies have investigated its mutant types. An increase in α-Syn and its phosphorylated forms may be associated with neuroinflammation and neurological deficits. These findings suggest the involvement of α-Syn in the response to ischemic stress and its role in the pathophysiology of neurodegenerative processes. As a result, concerning α-Syn, we can make a firm deduction that it increases. However, there is a need for more studies on its mutant types.

### Human Studies

Only two studies have explored the amount of α-Syn in humans. *Zhao* et al. spotted the amounts of oligomeric α-Syn in the red blood cell (RBC) by BCA assay using a spectrophotometer of PD patients with ischemic stroke patients. They investigated the role of α-Syn as a biomarker for diagnostic purposes. The levels of α-Syn were considerably greater in the ischemic group. There was no correlation between age and α-Syn levels. A high sensitivity of α-Syn was observed in differentiating ischemic patients from healthy controls (sensitivity = 63.7% and specificity = 9.6%). α-Syn can be regarded as a biomarker for the diagnosis of stroke (Zhao et al., 2016). These findings indicate that different forms of α-Syn can be detected in the blood of patients with cerebral ischemia. The levels of oligomeric and other subtypes of α-Syn were significantly greater in RBCs of ischemic patients. As a result, it can be used as a diagnostic biomarker for ischemic stroke.

## Therapeutic Implications of Alpha-Synuclein in Cerebral Ischemia

As previously mentioned, α-Syn is involved in the pathophysiology of ischemic disorders. Its amount tends to increase following ischemia. As a result, studies investigating treatment methods could lead to novel methods for **the** treatment of ischemic disorders. Here, we summarize studies involving treatments based on α-Syn.

First, three studies deduced certain medications that might act on α-Syn. *Ruzza* et al. used ceftriaxone to treat cerebral ischemia, as it is considered to be effective in some neurodegenerative disorders. It opposes glutamate-mediated toxicity and causes misfolded protein degeneration. As α-Syn plays a role in the pathology of PD, ceftriaxone has shown efficacy in the treatment of PD, and the efficacy of its interaction with α-Syn has been investigated. They indicated that it binds to α-Syn. In vitro, it blocks α-Syn polymerization. Therefore, it has neuroprotective effects in vitro and is considered a potential treatment option for various neurodegenerative diseases (Ruzza et al., 2014).

*Sato* et al. used telmisartan in the model of transient MCAO. Telmisartan is an angiotensin receptor blocker that protects against brain damage and neurodegeneration. They investigated its effect on oxidative stress caused by p-Syn accumulation in the mouse brain. They used stroke-resistant spontaneously hypertensive rats (SHRs-SRs). They reported that hypertension following stroke increased oxidative stress, with an accumulation of pain. They reported that the number of p-Syn-positive cells in the cerebral cortex and the hippocampus decreased in the treatment group (Sato et al., 2014). Fukui et al. performed the same experiment. They also reported that telmisartan reduces the accumulation of p-Syn caused by persistent hypertension (Fukui et al., 2014).

*Wang* et al. used propofol in cerebral ischemia. Propofol is neuroprotective in brain injury, but the mechanism is not clear. Neurotoxic aggregation of α-Syn was reduced following propofol administration. The neurological deficits and infarct area that occurred after ischemia were reduced. Gene expression was measured by Kyoto Encyclopedia of Genes and Genomes (KEGG); also, total α-Syn expression measured by immunoblotting was unchanged. Therefore, propofol might regulate α-Syn following transcription. It reduces the increase in autophagy following ischemia. These authors reported interactions between α-Syn and propofol and between α-Syn and mTOR, and between α-Syn and autophagy. They reported that its effect is related to the mammalian target of the rapamycin/ribosomal protein S6 kinase beta-1 signaling pathway, which inhibits autophagy (Wang et al., 2020).

*Xiong* et al. explored ketamine as a potential drug for treating cerebral ischemia, predicting its target via various databases and analyzing it using Gene Ontology (GO) and KEGG. They reported that one of the targets of ketamine is α-Syn. It binds to SNCA and inhibits the process of protein formation. Therefore, it decreases apoptosis and neural death and improves brain function. Ablation of the SNCA can be protective against cerebral ischemia (Xiong et al., 2022).

In conclusion, Ceftriaxone and telmisartan have neuroprotective effects by reducing α-Syn aggregation. Propofol decreased neurotoxic α-Syn accumulation and infarct size via the regulation of autophagy. Ketamine also binds to α-Syn, inhibiting protein formation and reducing neural apoptosis. Overall, medications targeting α-Syn can be used to treat ischemic disorders.

Second, one study researched αSyn-knockout or α-Syn-knockdown models. *Kim* et al. reported that knocking out or knocking down α-Syn decreases injury and improves neural function. They reported that the induction of phospho-Drp1, 3-nitrotyrosine, microtubule-associated protein 1 light chain 3 (LC-3), also caspase-3 was decreased in a α-Syn knocked-down model. These findings suggest that α-Syn has a role in autophagy, oxidative stress, and mitochondrial fragmentation. Additionally, knocking down PLK-2, which plays a role in S129 phosphorylation, improves recovery following ischemia, decreases infarction, and improves neural function (Kim et al., 2016). Overall, these findings indicate that α-Syn can be used as a therapeutic agent in cerebral ischemia.

Third, studies have focused on **the** use of microRNAs or siRNAs *to repress* α-Syn. *Kim* et al. injected miR-7a-5p locally or systemically to repress α-Syn to decrease brain damage following ischemia in a mouse model of MCAO. Decreased expression of miR-7 was observed post-ischemia. They proved that α-Syn is a miR-7 target and it has a binding site for αSyn. miR-7 suppresses α-Syn expression. Deleting the α-Syn gene eliminated miR-7 recovery and neuroprotection. Both intracerebral and intravenous administration of miR-7 caused improved cognitive and motor function and reduced infarct volume. These findings suggest that miR-7 can be used to decrease brain damage following ischemia. miR-7 administration affects mitochondrial dysfunction, apoptosis, oxidative stress, and autophagy, which are pathological ischemic markers (Kim et al., 2018). Similarly, *Mehta* et al. used miR-7 to treat cerebral ischemia. They explored motor function and infarction in double knockout miR-7 mice. The cognitive and motor functions of the miR-7^-/-^ healthy mice were similar to those of the miR^+/+^ healthy mice. However, miR-7^-/-^ mice with cerebral ischemia presented severe damage and also decreased motor function. Injecting miR-7 into miR-7^-/-^ mice restored neuroprotection and motor function. Overall, miR-7 averts α-Syn translation and reduces brain damage (Mehta et al., 2023). In another study, *Mehta* et al. evaluated the impact of the circular RNA Complementarity-Determining Region 1 antisense (CDR1as), which regulates miR-7. Both miR-7 and CDR1as are downregulated postischemia. The injection of an AAV9 vector harboring the CDR1as gene augmented its levels. The overexpression of CDR1as increased the level of miR-7 and repressed α-Syn induction poststroke. α-Syn induction following stroke is CDR1as/miR-7 dependent. It improved motor function and decreased neurological deficits. It decreased brain damage and the infarct area. Additionally, it decreased apoptosis, mitochondrial autophagy, and inflammation. CDR1as is considered neuroprotective because it protects miR-7 (Mehta et al., 2023). *Chelluboina* et al. injected siRNA intravenously to repress α-Syn. A ΑSyn knockdown decreased brain damage following ischemia and improved the recovery of motor function. One limitation of this treatment is the limited window of therapeutic opportunity for siRNAs. They reported that preventing α-Syn expression after ischemia can decrease damage and improve motor function, which was observed (Chelluboina et al., 2020). Overall, miRNAs and siRNAs can be used to target α-Syn and have shown potential in reducing ischemic brain damage. A lack of miR-7 resulted in severe brain damage; therefore, injection of miR-7a-5p mimic improved cognitive and motor functions. Additionally, the circular RNA CDR1as, which regulates miR-7, reduces α-Syn levels and improves recovery poststroke. Overall, these studies indicate that targeting α-Syn may effectively decrease ischemic injury.

In conclusion, certain medications that might target α-Syn, such as ceftriaxone, telmisartan, or ketamine, or repress α-Syn through mRNA, siRNA, or circular RNA, can be used as treatment methods for cerebral ischemia. However, because some of these treatment methods involve only one study, a firm conclusion cannot be drawn. More studies are needed in this regard to move from in vivo studies to clinical studies (Table 1).

**Table 1.** α-Synuclein in cerebral ischemia.

References	Ischemia method	Therapeutic method/regulators	Measurement method	Expression change	Possible mechanism
Koh et al. (2017)	pMCAO	–	Western blot	Decreased (after ischemia)	PI3/Akt, Bcl-2
Taguchi et al. (2020)	GBO	–	Immunohistochemistry	Coexpression decreases in the olfactory lobe	SPX2
Mehta et al. (2023)	tMCAO	miR-7	immunohistochemistry	Decreases (following treatment)	–
Ischimaru et al. (1998)	tBCAO	–	Immunohistochemistry	Increased (after ischemia)	–
Yu et al. (2004)	–	–	Western blot, Immunohistochemistry	Increased (after ischemia)	–
Yoon et al. (2006)	tBCAO	–	Immunohistochemistry, western blot	Increased (after ischemia)	–
Hu et al. (2006)	tCAO	–	Electrophoresis, mass spectrometry, and western blot	Increased (after ischemia)	dopamine
Unal-Cevik et al. (2011)	tMCAO	–	Immunohistochemistry, Western blot	All forms increased (after ischemia)	Oxidative stress
Kim et al. (2016)	tMCAO	Knock-out or siRNA-mediated knockdown	Western blot	Increased (after ischemia)	phosphor-Drp1,3-nitrotyrosine, LC-3, and caspase-3
Lohmann et al. (2022)	pMCAO	–	Immunohistochemistry, ELISA	Decreased monomer, increased aggregation	dopamine
Zhao et al. (2016)	Human Study	–	ELISA	Oligomeric increased (after ischemia)	–
Ruzza et al. (2014)	–	Ceftriaxone	spectroscopy	Decreased (following treatment)	–
Sato et al. (2014)	tMCAO	Telmisartan	Immunohistochemistry	Decreased (following treatment)	Oxidative stress
Kim et al. (2018).	tMCAO	miR-7	Western blot	Decreased (following treatment)	–
Wang et al. (2020)	tBACO	Propofol	Western blot, immunoblot	Unchanged monomer decreased aggregation (following treatment)	mTOR/S6K1
Xiong et al. (2022)	–	ketamine	Molecular docking	Inhibit	–
Chelluboina et al. (2020)	tMCAO	siRNA-mediated knockdown	Western blot	Decreased (following treatment)	–
Mehta et al. (2023)	tMCAO	CDR-1	Western blot, immunohistochemistry	Decreased (following treatment)	CDR-1/miR-7

GBO: global bilateral occlusion, t: transient, p: permanent, MCAO: middle cerebral artery occlusion, BCAO: bilateral carotid artery occlusion, CAO: coronary artery occlusion, miRNA: microRNA, siRNA: small interfering RNA, Complementarity-Determining Region 1, PI3K/Akt: Phosphoinositide 3-kinase/Protein Kinase B, Bcl-2: B-cell lymphoma 2, SPX2: Spexin 2, DRP1: Dynamin-related protein 1, S6K1:ribosomal protein S6 kinase 1, mTOR: mammalian target of rapamycin, LC3: microtubule-associated protein 1 light chain 3.

## Limitations and Future Directions

Research linking α-Syn to cerebral ischemia faces clear challenges. Most evidence comes from animal models like MCAO and BCAO, which do not fully reflect human stroke. The dual role of α-Syn—sometimes protective, sometimes harmful—remains uncertain, and human studies are limited, focusing mainly on peripheral biomarkers such as α-Syn in red blood cells.

Future work should emphasize longitudinal studies in humans, using imaging and molecular profiling to track α-Syn. Integrating transcriptomic and proteomic methods may uncover its regulatory networks, including interactions with miR-7 and CDR1as (Kim et al., 2018; Mehta et al., 2023). Clinical trials of α-Syn-targeting agents such as telmisartan, ceftriaxone, and propofol are needed to move findings into practice (Ruzza et al., 2014; Sato et al., 2014; Wang et al., 2020). A systems biology approach that considers genetic, epigenetic, and environmental factors will be crucial for understanding α-Syn’s complex role in cerebral ischemia.

## Conclusion

In conclusion, the intricate involvement of α-Syn in cerebral ischemia pathophysiology highlights its potential as a therapeutic target for mitigating ischemic brain injury. Further research into its molecular mechanisms and translational applications may significantly advance stroke treatment and improve patient outcomes.

## Data Availability

The figures in this manuscript were created using BioRender.com and are included in the article. Tables were manually compiled from published literature and are available upon request. No raw datasets were generated or analyzed during this review.
